# α-Gal antigen-deficient rabbits with *GGTA1* gene disruption via CRISPR/Cas9

**DOI:** 10.1186/s12863-022-01068-4

**Published:** 2022-07-11

**Authors:** Lina Wei, Yufeng Mu, Jichao Deng, Yong Wu, Ying Qiao, Kun Zhang, Xuewen Wang, Wenpeng Huang, Anliang Shao, Liang Chen, Yang Zhang, Zhanjun Li, Liangxue Lai, Shuxin Qu, Liming Xu

**Affiliations:** 1 National Institutes for Food and Drug Control, Beijing, 102629 China; 2 School of Materials Science and Engineering, Southwest Jiaotong University, Chengdu, 611756 China; 3 Département de Biologie Médicale, Université du Québec à Trois-Rivières, Trois-Rivières, QC G9A5H7 Canada; 4 Beijing YiSai Biotechnology Co., Ltd, Beijing, 100176 China; 5 Guangzhou ZhongDa Medical Equipment Co., Ltd., Guangzhou, 511458 China; 6 Jilin Provincial Key Laboratory of Animal Embryo Engineering, College of Animal Science, Jilin University, Changchun, 130062 China; 7 Key Laboratory of Regenerative Biology, Chinese Academy of Science, and Guangdong Province Key Laboratory of Stem Cells and Regenerative Medicine, South China Institute for Stem Cell Biology and Regenerative Medicine, Guangzhou Institutes of Biomedicine and Health, Guangzhou, 510530 China

**Keywords:** α-Gal antigen, *GGTA1* gene, Gal antigen-deficient rabbit, Immunogenicity, CRISPR/Cas9, Implant response

## Abstract

**Background:**

Previous studies have identified the carbohydrate epitope Galα1–3Galβ1–4GlcNAc-R (termed the α-galactosyl epitope), known as the α-Gal antigen as the primary xenoantigen recognized by the human immune system. The α-Gal antigen is regulated by galactosyltransferase (GGTA1), and α-Gal antigen-deficient mice have been widely used in xenoimmunological studies, as well as for the immunogenic risk evaluation of animal-derived medical devices. The objective of this study was to develop α-Gal antigen-deficient rabbits by *GGTA1* gene editing with the CRISPR/Cas9 system.

**Results:**

The mutation efficiency of *GGTA1* gene-editing in rabbits was as high as 92.3% in F0 pups. Phenotype analysis showed that the α-Gal antigen expression in the major organs of F0 rabbits was decreased by more than 99.96% compared with that in wild-type (WT) rabbits, and the specific anti-Gal IgG and IgM antibody levels in F1 rabbits increased with increasing age, peaking at approximately 5 or 6 months. Further study showed that *GGTA1* gene expression in F2-edited rabbits was dramatically reduced compared to that in WT rabbits.

**Conclusions:**

α-Gal antigen-deficient rabbits were successfully generated by *GGTA1* gene editing via the CRISPR/Cas9 system in this study. The feasibility of using these α-Gal antigen-deficient rabbits for the in situ implantation and residual immunogenic risk evaluation of animal tissue-derived medical devices was also preliminarily confirmed.

**Supplementary Information:**

The online version contains supplementary material available at 10.1186/s12863-022-01068-4.

## Background

Animal tissue-derived biomaterials have been widely used in wound repair, tissue and organ regeneration and other medical applications due to their good biocompatibility and ability to induce tissue regeneration relative to synthetic materials. However, the application of animal tissue-derived biomaterials to the human body carries a potential risk of immune rejection or undesired/unexpected immune response and inflammation, which directly affects the safety and effectiveness of these materials [1]. Wild-type (WT) experimental animals are traditionally used to evaluate the biological safety of medical devices. For the safety evaluation of animal tissue-derived medical devices with respect to features such as immunogenicity and host implant response evaluation, it is obviously unreasonable to use WT experimental animals. Because of the different sensitivities of experimental animals and human beings to xenoantigens from animals, it is impossible to objectively evaluate the safety risk of animal tissue-derived medical devices implanted into human beings through experiments in these models.

Previous studies have identified the primary xenoantigen as the galactosyl-containing epitope Galα1–3Galβ1–4GlcNAc-R (termed the α-galactosyl epitope), also known as the α-Gal antigen, which is mainly regulated by galactosyltransferase (GGTA1) [2]. This antigen is expressed in all animals except apes, baboons and old-world monkeys but is not expressed in humans [3]. However, under the stimulation of the intestinal flora, which also express α-Gal antigen, the human body generates high levels of anti-Gal antibodies [4]. This is the main reason for the hyperacute immune rejection of animal tissues and organs that are implanted in the human body without prior antigen removal [5].

The raw materials for animal tissue-derived medical devices are primarily derived from pigs, cattle, horses, and even rats. However, the most commonly used experimental animals, such as mice, rats and rabbits, all express the α-Gal antigen, and therefore are not susceptible to significant immune rejection reaction caused by residual α-Gal antigen in the animal tissue-derived implanted materials. As a consequence, the immunogenicity risk of materials derived from animal tissues and implanted into the human body cannot be reasonably evaluated by using WT experimental animals.

Currently, the animal models of α-Gal antigen deficiency described in the literature are mostly *GGTA1* knockout mice and pigs [6–10]. α-Gal antigen-deficient mice have been used in many studies to evaluate the immunogenicity of animal tissues, organs and animal tissue-derived biomaterials [11, 12]. Our group also developed α-Gal antigen-deficient mice through *GGTA1* knockout, and these model animals have been widely used in the immunogenic risk evaluation of animal tissue-derived medical devices [13–17].

For implantable animal tissue-derived medical devices, such as biological corneas, bone xenografts, and biological dura meshes, testing the host response to such implanted medical devices requires evaluation of in situ implantation in a reasonable model animal. Mice are the most widely used laboratory animals, but they are too small for in situ implantation studies such as xeno-corneal implantation or xeno-bone implantation. Model pigs are most commonly used for the investigation of xeno tissue or organ transplantation [18]. However, the long breeding time and high costs limit the use of gene-edited pigs as experimental animals. Rabbits have traditionally been used as laboratory animals and are widely used in medical device implantation tests. However, no α-Gal antigen-deficient rabbit models have been reported thus far. The objective of this study was to develop a novel α-Gal antigen-deficient rabbit model with the *GGTA1* gene edited via CRISPR/Cas9 that can be used for implantation tests and for evaluating the residual immunogenic risk of animal tissue-derived medical devices.

### ResultsCRISPR/Cas9-mediated gene targeting of *GGTA1* in zygotes

The rabbit *GGTA1* gene information was gathered from the NCBI website and an automated bioinformatic gene prediction method (Gnomon), and the spliced sequence was obtained (gene ID: LOC100348435). The coding sequence of rabbit N-acetyllactosaminide alpha-1,3-galactosyl transferase (*GGTA1*) was analyzed through information comparison and screening and found to include Exon 1: 16–286, Exon 2: 34958–35,072, Exon 3: 41258–41,346, Exon 4: 48906–48,941, Exon 5: 51613–51,678, Exon 6: 52163–52,279, Exon 7: 59218–59,355, and Exon 8: 63151–63,844. Exon 8, with a full length of 694 bp, was the longest and exhibited a 99% match with the known, recognized and validated mouse *GGTA1* domain (exon 9); therefore, it was selected as the rabbit *GGTA1* functional domain.

**Fig. 1:**
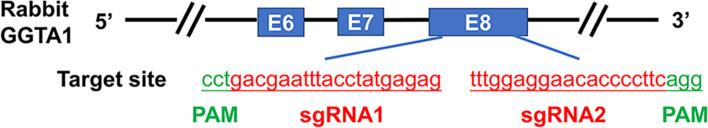
Schematic diagram of sgRNA targeting the *GGTA1* gene loci

To disrupt the *GGTA1* gene in rabbits, two sgRNAs targeting the CDS of *GGTA1* were designed (Fig. 1 and Table 1).

**Table 1:** Oligos synthesized for *GGTA1* sgRNAs

Target gene	Target site	PAM	Oligonucleotide1	Oligonucleotide2
*GGTA1*-sgRNA1	CTCTCATAGGTAAATTCGTC	AGG	TAGGCTCTCATAGGTAAATTCGTC	AAACGACGAATTTACCTATGAGAG
*GGTA1*-sgRNA2	TTTTGGAGGAACACCCCTTC	AGG	TAGGTTTTGGAGGAACACCCCTTC	AAACGAAGGGGTGTTCCTCCAAAA

To clone the sgRNA sequence into the pUC57-T7-gRNA vector, a *Bbs*I enzyme cut site was added next to the complementary DNA oligonucleotides (Table 1). To determine the efficiency of *GGTA1* gene modification by the CRISPR/Cas9 system, in vitro transcribed mRNA from Cas9 and sgRNAs was microinjected into zygotes, and the zygotes were cultured to the blastocyst stage.

### Generation of *GGTA1* gene-edited rabbits

A total of 224 microinjected zygotes (pronuclear stage) were transferred into the oviducts of 4 surrogate rabbits (Table 2). After 30 days of gestation, three recipient mothers gave birth to 15 rabbit pups, 2 of which were born dead and not counted in Table 2. One receptor rabbit failed to become pregnant; it is possible that this receptor was not in estrus.

**Table 2:** Generation of *GGTA1-*edited rabbits via the CRISPR/Cas9 system

Recipients	gRNA/Cas9 mRNA (ng/μL)	Embryos transferred	Pregnancy	Pups obtained (% transferred)	Pups with mutations (%)
1	40/200	60	Yes	4 (6.7%)	4 (100%)
2	40/200	54	Yes	5 (9.3%)	5 (100%)
3	40/200	52	No	0 (0%)	0 (0%)
4	40/200	58	Yes	4 (6.9%)	3 (75.0%)
Total	/	224	/	13 (5.8%)	12 (92.3%)

### Genotypes of the *GGTA1* gene-edited F0 rabbits

**Fig. 2:**
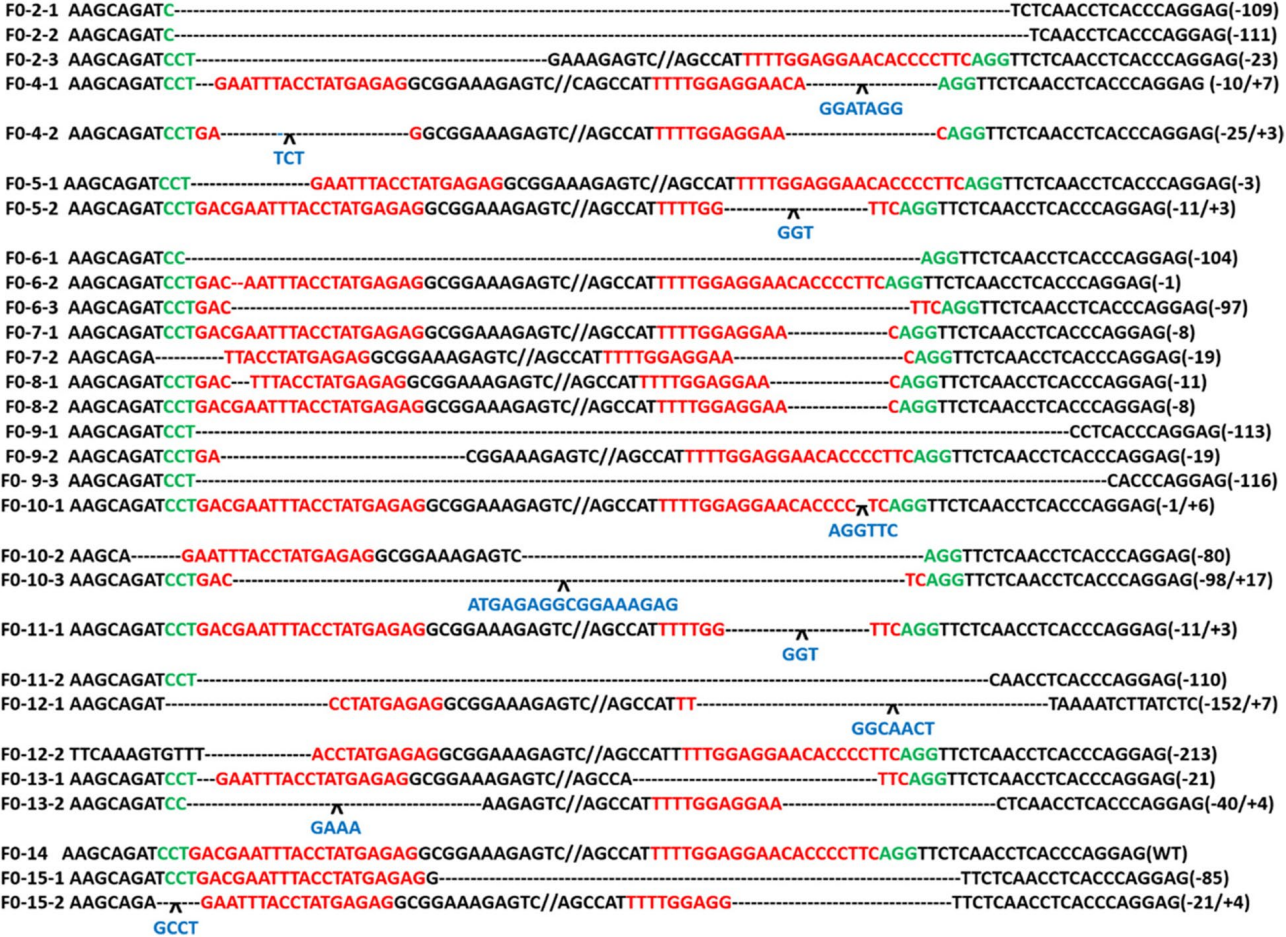
T-cloning and Sanger sequencing in 13 pups (F0 rabbits) with *GGTA1* gene modification. F0–1 and F0–3 are not shown because they were born dead. The sgRNA sequences are highlighted in red, PAM sequences in green and insertions in blue. Deletion “−”; insertion: “+”

Genomic DNA from the ears of 13 obtained live *GGTA1* gene-edited F0 rabbit pups was isolated and subjected to PCR and sequencing for mutation detection. The results of Sanger sequencing (Fig. 2) showed that the genotype of the 14th rabbit pup (F0–14) was WT, while the remaining 12 rabbit pups had mutated *GGTA1*. As shown in Fig. 2 and Table 2, the mutation efficiency of F0 *GGTA1* gene-edited rabbits was as high as 92.3% in live F0 pups. These results indicated that the dual sgRNA-directed CRISPR/Cas9 system efficiently mutated rabbit *GGTA1* in this study. However, it cannot be ignored that several of the pups (F0–6, F0–9, and F0–10) were chimeras.

### Off-target analysis of F0 *GGTA1* gene-edited rabbits

**Fig. 3:**
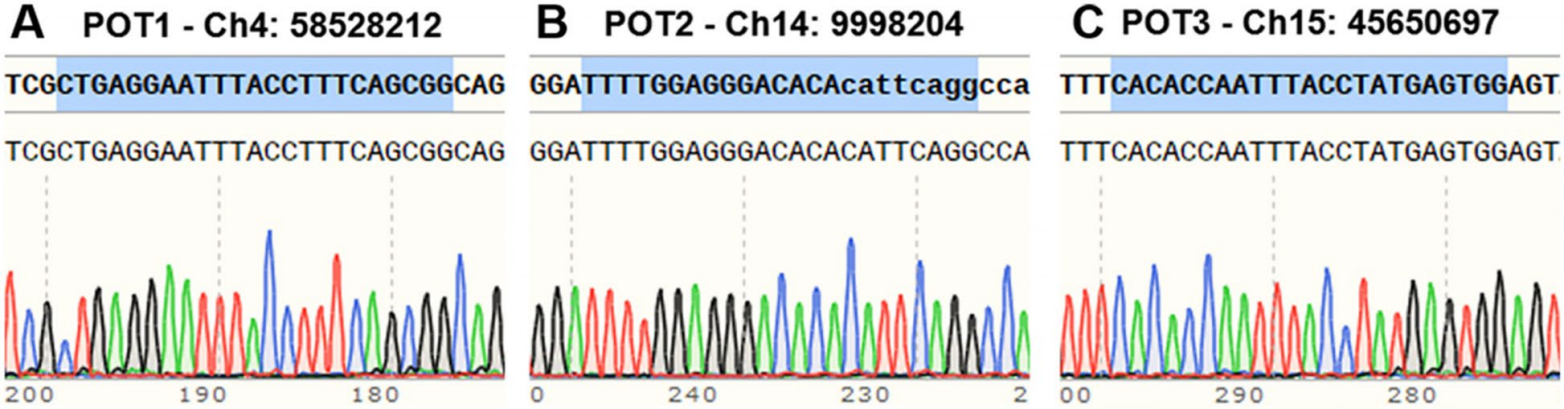
Off-target detection in the F0 generation of *GGTA1-*edited rabbits. Chromatogram sequence analysis of two potential off-target sites (POTS) for sgRNA1 (**A**, **B**) and one POTS for sgRNA2 (**C**) using PCR products in founders

Off-target effects are a major concern when using the CRISPR/Cas9 system. To test whether off-target mutagenesis occurred in the *GGTA1-*edited rabbits, we performed Sanger sequencing on PCR products from 4 potential off-target sites (POTS) with 3 mismatches: 3 POTS for sgRNA1 and 1 POTS for sgRNA2 (Chr3: 121750284, Chr4: 58528212, Chr15: 45650697, and Chr14: 9998204). Unfortunately, the PCR of one POTS (Chr3: 121750284) failed, possibly because this POTS was rich in N. Without an accurate reference genome, proper primers for the verification of this site could not be designed. The results of the remaining 3 POTS, shown in Fig. 3, demonstrated that no mutation had occurred, indicating that the Cas9/sgRNA system most likely did not induce undesirable off-target effects in *GGTA1*-edited rabbits.

### Heritability of the *GGTA1* mutations in gene-edited rabbits

**Fig. 4:**
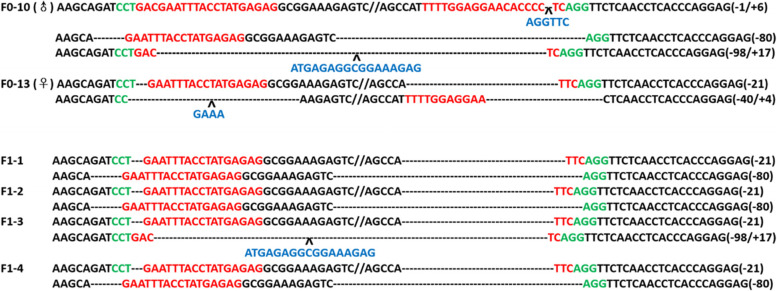
T-cloning and Sanger sequencing analysis of F1 pups (F0–10 mated with F0–13). Deletion: “-”, insertion: “+”

To study whether the induced deletions or indels were heritable, the genotypes of the F1 pups (F0–10 mated with F0–13) were determined by PCR and T-cloning Sanger sequencing. As shown in Fig. 4, all of the F1 rabbits had the mutation. Because F0–10 was a chimera, the F1 rabbits exhibited 2 different genotypes. The genotype of F1–3 was obviously different from those of F1–1, F1–2, and F1–4.

### α-Gal antigen expression of the F0 *GGTA1* gene-edited rabbits

**Fig. 5:**
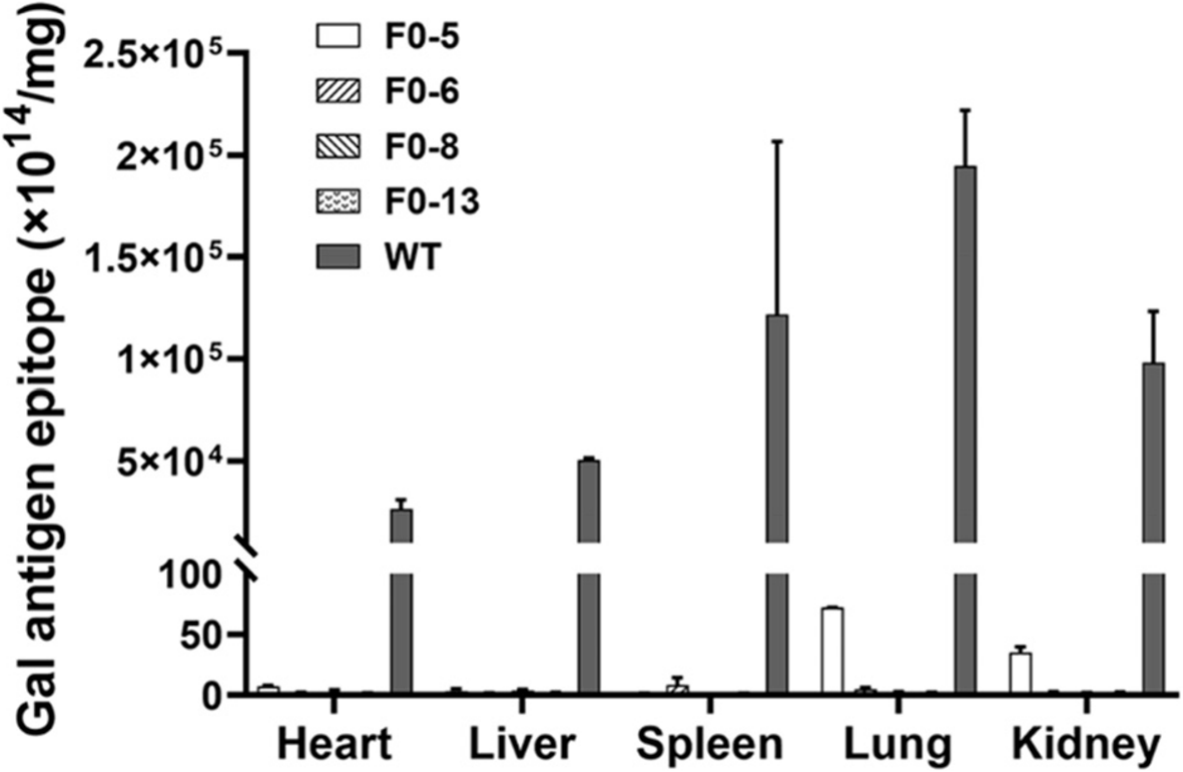
α-Gal antigen epitope expression in the major organs of F0 rabbits and WT rabbits. F0–5, F0–6, F0–8, and F0–13 were the 4 F0 *GGTA1-*edited rabbits examined

To investigate the phenotype of the *GGTA1* gene-edited rabbits, the expression level of α-Gal antigen, which is mainly regulated by GGTA1, was determined via an inhibition enzyme-linked immune sorbent assay (ELISA). α-Gal antigen expression was detected in major organs, namely, the heart, liver, spleen, lung, and kidney, of 4 F0 *GGTA1* gene-edited rabbits and WT rabbits (Fig. 5).

The results showed that α-Gal antigen was almost completely absent in the different organs of different *GGTA1* gene-edited rabbits. Specifically, compared with that in WT rabbits, the relative expression level of α-Gal antigen in *GGTA1* gene-edited rabbits was decreased by more than 99.97% (F0–5) in the heart, 99.99% (F0–8) in the liver, 99.99% (F0–6) in the spleen, 99.96% (F0–5) in the lung, and 99.96% (F0–5) in the kidney. The data presented here suggested that α-Gal antigen-deficient rabbits were successfully obtained by *GGTA1* gene editing via the CRISPR/Cas9 system.

### Anti-Gal antibody levels of the F1 *GGTA1* gene-edited rabbits

To further study the phenotypes of *GGTA1* gene-edited rabbits, which were confirmed to be α-Gal antigen deficient, the specific anti-Gal IgG and anti-Gal IgM antibody levels in 3 *GGTA1* gene-edited F1 pups were continuously monitored and determined by ELISA. Theoretically, given the lack of α-Gal antigen expression, the animals should express anti-Gal antibodies.

**Fig. 6:**
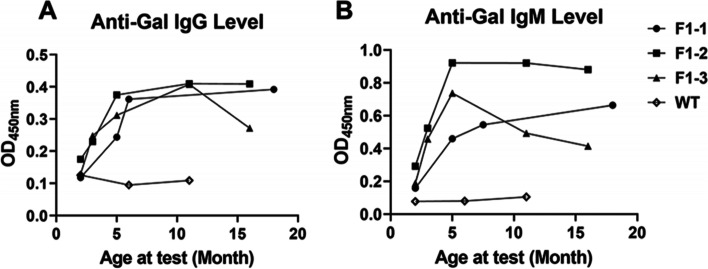
The anti-Gal IgG (**A**) and anti-Gal IgM (**B**) antibody levels in 3 F1 rabbits, F1–1, F1–2, and F1–3, compared with WT rabbits

According to the ELISA results, the optimal density (OD_450nm_) of all the samples was positively related to serum dilution (1:400, 1:800, 1:1600) and had good linearity (data not shown). The OD_450nm_ of all the samples at the same dilution (1:400) is shown in Fig. 6. The results showed that the anti-Gal IgG and IgM antibodies were absent from WT rabbits. In contrast, in the 3 *GGTA1* gene-edited F1 pups shown to be α-Gal deficient by red blood cell detection (data not shown), the anti-Gal IgG and IgM antibody levels increased with age and peaked at approximately 5 to 6 months.

However, the trends of the increases in anti-Gal antibodies were different in different Gal-deficient pups. As shown in Fig. 6, both anti-Gal IgG and IgM antibodies decreased from their peak levels (at 11 months and 6 months, respectively) in rabbit F1–3 but were maintained at high levels in rabbits F1–1 and F1–2, indicating that there are individual variations in the anti-Gal IgG and IgM antibody levels among the *GGTA1-*edited rabbits.

### *GGTA1* gene expression in F2 *GGTA1* gene-edited rabbits

**Fig. 7:**
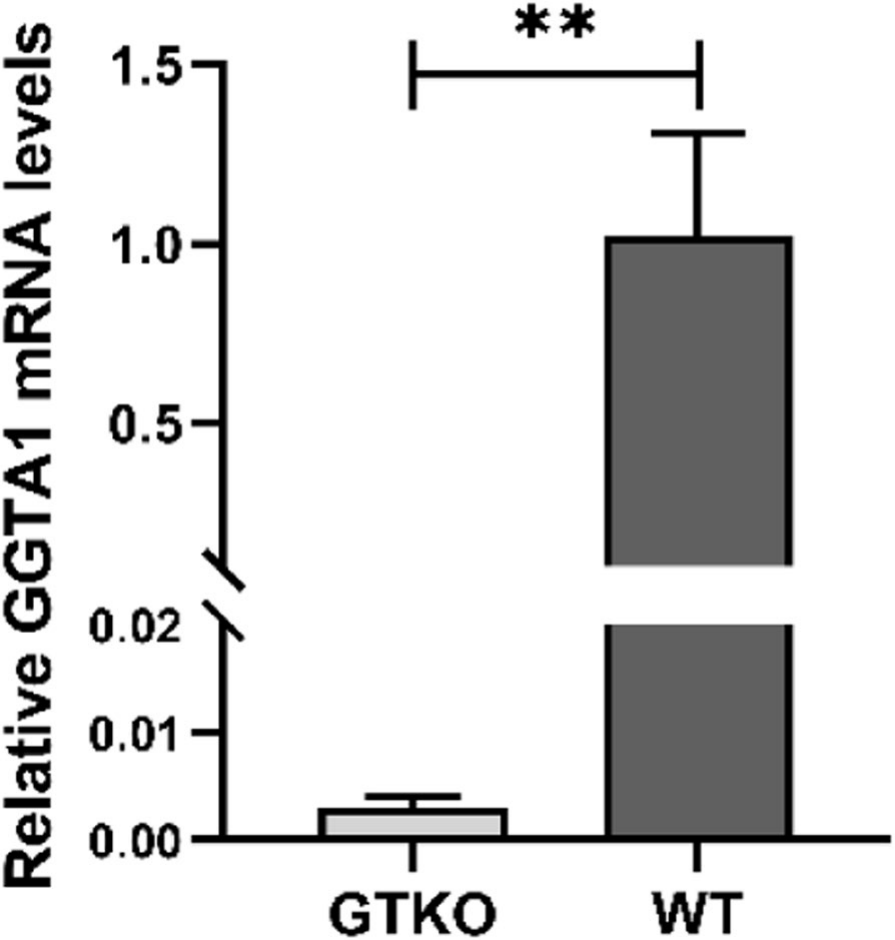
*GGTA1* gene expression levels in lung tissue of 3 F2 *GGTA1* gene-edited rabbits compared with 3 WT rabbits. A probability of *P* < 0.05 was considered statistically significant. GTKO: F2 *GGTA1* gene-edited rabbits; **, *P* < 0.01

To explore the mechanisms of α-Gal antigen deficiency and anti-Gal antibody presence in *GGTA1* gene-edited rabbits, we used qRT–PCR to assay *GGTA1* gene expression in the lung tissue of 3 F2 *GGTA1* gene-edited rabbits and 3 WT rabbits. These F2 rabbits were homozygous for a 21 bp deletion at the *GGTA1* gene site; the genotypes are shown in Fig. 4. The results showed that *GGTA1* expression in edited rabbits (0.003 ± 0.001) was dramatically reduced compared to that in WT rabbits (1.000 ± 0.245, *P* < 0.01), as shown in Fig. 7. This might result in significant downregulation of the GGTA1 protein level and further α-Gal antigen deficiency and anti-Gal antibody presence in *GGTA1* gene-edited rabbits.

### Anti-Gal antibody levels of F2 *GGTA1* gene-edited rabbits after in situ implantation

To preliminarily investigate the feasibility of using *GGTA1* gene-edited rabbits for in situ implantation and evaluating the residual immunogenic risk of animal tissue-derived medical devices, xenogeneic corneal matrix (pig sourced) and bone substitutes (bovine sourced) without decellularization and xenoantigen removal were implanted into two F2 *GGTA1* gene-edited homozygous rabbits with a 21 bp deletion.

**Fig. 8:**
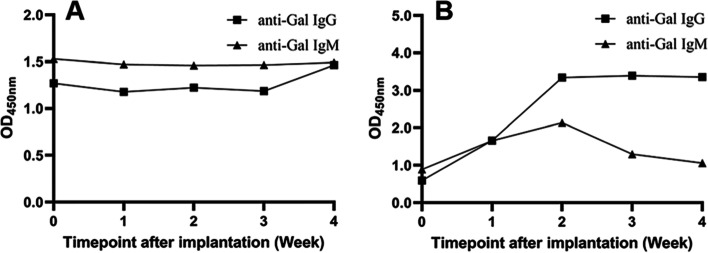
The anti-Gal antibody level of F2 rabbits after in situ implantation of (**A**) pig corneal matrix and (**B**) bovine bone substitute

The anti-Gal antibody levels of the two *GGTA1* gene-edited rabbits were evaluated before implantation and weekly (up to 4 weeks) after implantation. The OD_450 nm_ of the samples at a 1:400 dilution for the pig corneal matrix and a 1:500 dilution for the bovine bone substitute are shown in Fig. 8.

According to Fig. 8, no obvious trends were observed for anti-Gal IgG and IgM antibodies in the rabbit implanted with pig corneal matrix after implantation (Fig. 8 A), while the anti-Gal IgG and IgM antibodies in the rabbit implanted with bovine bone substitutes increased with time after implantation, especially during the first two weeks (Fig. 8 B). Thereafter, the anti-Gal IgG antibody level remained at a similar level from the 2nd week to the 4th week, while the anti-Gal IgM antibody level decreased continually until the 4th week.

These results showed that *GGTA1*-edited rabbits generated different levels of specific antibodies after implantation of different materials in different body parts, indicating that α-Gal antigen-deficient rabbits may be feasible for use for in situ implantation and evaluation of residual immunogenic risk in animal tissue-derived medical devices.

## Discussion

The gene sequencing results showed that the targeted exon 8 fragment of the *GGTA1* gene of the rabbits was successfully edited, except in the F0–14 pup, which did not have the *GGTA1* gene mutation. The expression of the α-Gal antigen was reduced by 99.96% or more in the major organs (heart, liver, spleen, lung, and kidney) in all of the F0 pups. This report is the first to elucidate α-Gal antigen expression after *GGTA1* gene editing in rabbits.

Due to the technical shortcomings of CRISPR/Cas9, the gene bases affected in these animals were variable in location and/or length. All the F0 pups carried different GGTA1 gene mutations, and some pups were chimeras (F0–6, F0–9, and F0–10). Therefore, further selection and purification to breed uniform populations (i.e., with the same *GGTA1* gene mutations) are necessary and are ongoing in our laboratory.

Previous studies have shown that aside from GGTA1, a second enzyme, isoglobotrihexosylceramide synthase (iGb3S), is able to synthesize α-Gal antigen in mice and rats [7, 19]. Our previous study showed that α-Gal epitope expression was reduced by 100% in the major organs of *GGTA1/iGb3S* double knockout mice compared with their WT counterparts [14]. Further research is needed to determine whether *iGb3S* contributes to the residual α-Gal antigen expression (less than 0.04%) observed after *GGTA1* gene editing in our rabbits.

Analyses of the major organs of WT rabbits indicated that α-Gal antigen expression was significantly higher in the spleen and lung than in other organs (Fig. 5), which is also consistent with our previous study in *iGb3S*-deficient mice [20]. This is the first report confirming the α-Gal antigen expression patterns in different organs in rabbits, and this characteristic may be conserved among different species.

In this study, it was confirmed that WT rabbits express α-Gal antigen (Fig. 5) and lacked anti-Gal antibody expression in the serum (Fig. 6). In contrast, the 3 *GGTA1* gene-edited F1 pups were almost Gal-deficient, and their levels of anti-Gal antibodies increased with increasing age and peaked at approximately 5 to 6 months (Fig. 6). To more fully characterize the trends of the anti-Gal antibodies in a larger *GGTA1* gene-edited rabbit population, further investigations involving more pups are needed.

One reason for the different anti-Gal antibody trends over time of the two F2 *GGTA1* gene-edited rabbits after in situ implantation may be that the Gal-antigen content of the cornea (5.4 × 10^11^ epitopes/mg) is lower than that of the bone substitute (14.8 × 10^11^ epitopes/mg); as a result, the latter could stimulate the α-Gal antigen-deficient rabbits to produce higher levels of anti-Gal antibodies. The other reason may be that the cornea is immune privileged, so the immune reaction after implantation at this site is relatively minor [21]. Nevertheless, the above results provide preliminary confirmation that *GGTA1* gene-edited Gal-antigen-deficient rabbits can be used for in situ implantation and the evaluation of residual immunogenic risk from animal tissue-derived medical devices. Aside from anti-Gal antibodies, other humoral immunity factors, along with cellular immunity and local histopathology, should be further investigated to achieve comprehensive residual immunogenic risk evaluation of animal tissue-derived medical devices in these Gal-deficient rabbits. These analyses are ongoing in our laboratory.

## Conclusions

In this study, α-Gal antigen-deficient rabbits were successfully generated by *GGTA1* gene editing with the CRISPR/Cas9 system. The α-Gal antigen almost disappeared, and the anti-Gal antibody levels increased with age, in these *GGTA1* gene-edited rabbits. Moreover, the feasibility of using the α-Gal antigen-deficient rabbits generated in this study for in situ implantation and residual immunogenic risk evaluation of animal tissue-derived medical devices was preliminarily confirmed.

### MethodsCas9 mRNA and *GGTA1* sgRNA preparation

To target rabbit *GGTA1*, single guide RNAs (sgRNAs) were designed using online tools (http://crispr.mit.edu/). The gene targeting schematic is shown in Fig. 1. After comparative analysis, two target sequences were selected from the *GGTA1* functional region (in Exon 8). The sgRNA recognition sequence and the oligonucleotide chain are shown in Table 1.

The two complementary targeting sequence DNA oligonucleotides designed above were annealed at 95 °C for 5 minutes to synthesize double-stranded DNA; then, the double-stranded DNA was cloned into the *Bbs*I-linearized pUC57-T7 vector (gene ID 51306). The recombinant vector (pUC57-T7-*GGTA1*/sgRNA) was subsequently amplified with T7 primers (T7-F: 5`-GAAATTAATACGACTCACTAT A-3` and T7-R: 5`-AAAAAAAGCACCGACTCGGTGCCAC-3`). The gRNA PCR products were transcribed using the MAXIscript T7 kit (Ambion) and purified by the miRNeasy Mini Kit (Qiagen) according to the manufacturer’s instructions.

### Microinjection and embryo transfer

Female New Zealand White rabbits (6–8 months old) were super ovulated with FSH (50 IU) 6 times at intervals of 12 h. After the last injection, the female rabbits were mated with male rabbits. The females then received an injection of 100 IU human chorionic gonadotropin (HCG). At 18 h post-HCG injection, the female rabbits were euthanized, and their oviducts were flushed with 5 mL DPBS-BSA for zygote collection. Rabbit embryos at the pronuclear stage (approximately 18–20 h post mating) were collected and transferred into oocyte manipulation medium, which contained 9.5 g of TCM-199, 0.05 g of NaHCO_3_ (Sigma, S4019), 0.750 g of HEPES (Sigma, H3784), 0.05 g of penicillin, 0.06 g of streptomycin, 1.755 g of NaCl, 3.0 g of BSA, and 1 L of Milli-Q H_2_O. A mixture of Cas9 and sgRNA mRNA (200 ng/μL and 40 ng/μL, respectively) was microinjected into the embryo cytoplasm to edit the *GGTA1* gene.

The microinjected embryos were transferred into EBSS medium for short-term culture in a humid chamber at 38.5 °C and 5% carbon dioxide (CO_2_). Approximately 50–60 microinjected embryos were transferred into the oviduct of a recipient rabbit.

### Gene mutation detection by PCR in F0 and F1 pups

Genomic DNA was isolated from ear tissue samples from WT and *GGTA1-*edited rabbit pups using the TIANamp Genomic DNA Kit (TIANGEN). The DNA was amplified with 2× Taq Plus Master Mix (TIANGEN), and the PCR primers used to detect mutation were as follows: F-5’TGGAGGAGTTCATAACATCTGC-3′, and R-5’TGCTGGGATTATCATATAGGCCT-3′. The PCR products were purified and cloned into the pGM-T vector (TIANGEN). The colonies were picked and analyzed by Sanger sequencing to confirm the exact gene mutations.

### Off-target assay

The POTS of the two sgRNAs were predicted using a CRISPR design tool (http://www.rgenome.net/cas-offinder). There were no potential off-target sites (POTS) with only 1 or 2 mismatches. Three POTS for sgRNA1 and one POTS for sgRNA2, all of which had 3 mismatches (Chr3: 121750284, Chr4: 58528212, Chr15: 45650697, and Chr14: 9998204), were predicted and analyzed for site-specific cleavage by the CRISPR/Cas9 system. The PCR products of the POTS regions were also sequenced by Sanger sequencing.

### α-Gal antigen determination

The α**-**Gal antigen expression level was determined using a commercially available α-Gal antigen detection kit (Sanyao Science & Technology Co., Ltd., Beijing) via an inhibition enzyme-linked immune sorbent assay (ELISA). The specific experimental procedures are the same as those described in our previous publication [22] and the Chinese medical device industry standard YY/T 1561–2017 (Tissue engineering medical device products - Remnant α-Gal antigen determination in scaffold materials utilizing animal tissues and their derivatives).

### Anti-gal antibody analysis

The anti-Gal IgG and anti-Gal IgM antibody levels in *GGTA1*-edited rabbits were determined by ELISA with Gal-BSA (Dextra Laboratories Ltd., NGP0203) as a solid-phase antigen to capture the specific anti-Gal IgG and anti-Gal IgM and horseradish peroxidase (HRP)-conjugated goat anti-rabbit IgG (Abcam, ab6721) and IgM (Abcam, 97,195) secondary antibodies. Tetramethylbenzidine (TMB) HRP substrate buffer was then added to each well and incubated for 15 min at 37 °C. Finally, 10% H_2_SO_4_ was added to each well to stop the reaction, and the optical density was read at 450 nm using a microplate reader.

Briefly, based on preliminary data, 2 μg/mL Gal-BSA in carbonate buffer solution (pH 9.5 ~ 9.6) was selected to coat 96-well plates (100 μL/well) with incubation for 2 h at 37 °C. HSA (human serum albumin, 1%) in PBS was added and incubated for 2 h at 37 °C to block the nonspecific binding sites (200 μL/well).

Diluted serum (1:400, 1:800, 1:1600) collected from 3 F1 *GGTA1* gene-edited pups and 15 WT rabbits at different months of age (2 ~ 18 months for *GGTA1* gene-edited pups; 2, 6, and 11 months for WT pups) were added as the primary Abs and incubated for 2 h at 37 °C. Diluted serum of the F2 *GGTA1* gene-edited rabbit after in situ implantation collected at different weeks, 1:400 and 1:500 separately for pig cornea matrix and bovine bone substitute, were also added as primary Abs and incubated for 2 h at 37 °C. Diluted goat anti-rabbit IgG/IgM-HRP (1:16000) was loaded as a secondary antibody and incubated for 1 h at 37 °C. Between the above steps, 5 washes with 0.05% Tween-20 were performed.

### Quantitative real-time RT–PCR (qRT–PCR)

The protocol for RNA extraction was as described previously. The primers used for qRT–PCR of GGTA1 and GAPDH were F-5’TTTACCTATGAGAGGCGGAAAG-3′, R-5′ GAGGTTGAGAACCTGAAGGG-3′, and F-5’CACTTCGGCATTGTGGAG-3′, R-5’GAGGCAGGGATGATGTTCT-3′, respectively. *GGTA1* gene expression is presented as the mean ± SEM with normalization to the amount of GAPDH mRNA, as analyzed by the 2^−ΔΔCT^ formula and GraphPad Prism software (T test). A probability of *P* < 0.05 was considered statistically significant.

### Corneal matrix and bone substitute for in situ implantation

The pig-sourced cornea matrix and the bovine-sourced bone substitutes without decellularization and xenoantigen removal were provided by Guangzhou ZhongDa Medical Equipment Co., Ltd. and Guanhao Biotech Co., Ltd. separately. The process of preparing these two animal-derived materials from fresh raw tissues included physical cutting, defatting, and packaging sterilization.

One piece of corneal matrix was implanted into the left eye of one F2 *GGTA1* gene-edited rabbit (male, 10 months old at implantation), and two pieces of bone substitute (bovine source) were implanted into both condyles of the femur of the other F2 rabbit (female, 12 months old at implantation). These two F2 rabbits have the same F1 parents. The study protocol was approved by the Institutional Animal Care and Use Committee of the National Institutes for Food and Drug Control (NIFDC).

## Supplementary Information

**Additional file 1.**

## Data Availability

Genotypes for all mutant *GGTA1* rabbits were deposited at the Sequence Read Archive (SRA) under the project PRJNA784734.
